# Neoadjuvant botensilimab plus balstilimab response pattern in locally advanced mismatch repair proficient colorectal cancer

**DOI:** 10.1038/s41388-023-02835-y

**Published:** 2023-09-21

**Authors:** Pashtoon Murtaza Kasi, Manuel Hidalgo, Mehraneh D. Jafari, Heather Yeo, Lea Lowenfeld, Uqba Khan, Alana T. H. Nguyen, Despina Siolas, Brandon Swed, Jini Hyun, Sahrish Khan, Madeleine Wood, Benjamin Samstein, Juan P. Rocca, Allyson J. Ocean, Elizabeta C. Popa, Daniel H. Hunt, Nikhil P. Uppal, Kelly A. Garrett, Alessio Pigazzi, Xi Kathy Zhou, Manish A. Shah, Erika Hissong

**Affiliations:** 1 Department of Oncology/Hematology, New York Presbyterian/Weill Cornell Medicine New York, New York, NY 10021 USA; 2 Department of Surgery, New York Presbyterian/Weill Cornell Medicine New York, New York, NY 10021 USA; 3 Department of Population Health Sciences, Weill Cornell Medicine, New York, NY 10021 USA; 4 Department of Pathology and Laboratory Medicine, New York Presbyterian/Weill Cornell Medicine, New York, NY 10021 USA

**Keywords:** Biologics, Colorectal cancer

## Abstract

In patients with locally advanced cancer without distant metastases, the neoadjuvant setting presents a platform to evaluate new drugs. For mismatch repair proficient/microsatellite stable (pMMR/MSS) colon and rectal cancer, immunotherapy has shown limited efficacy. Herein, we report exceptional responses observed with neoadjuvant botensilimab (BOT), an Fc-enhanced next-generation anti–CTLA-4 antibody, alongside balstilimab (BAL; an anti-PD-1 antibody) in two patients with pMMR/MSS colon and rectal cancer. The histological pattern of rapid immune response observed (“*inside-out*” (serosa-to-mucosa) tumor regression) has not been described previously in this setting. Spatial biology analyses (RareCyte Inc.) reveal mechanisms of actions of BOT, a novel innate-adaptive immune activator. These observations have downstream implications for clinical trial designs using neoadjuvant immunotherapy and potentially sparing patients chemotherapy.

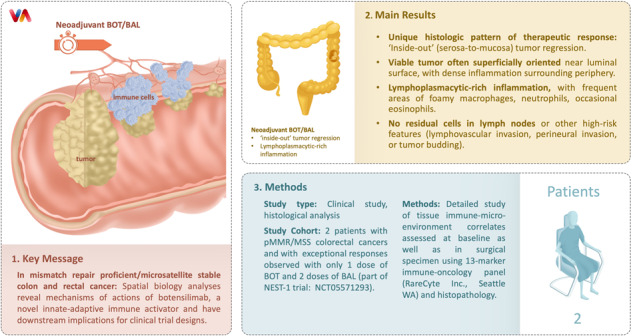

## Introduction

Amongst many advances, recognition of the *neoadjuvant* setting as potentially conducive to use of immunotherapy is notable [1], while representing a window of opportunity to efficiently evaluate new drugs [2, 3].

Botensilimab (BOT) is an Fc-enhanced next-generation anti– cytotoxic T-lymphocyte antigen 4 (CTLA-4) antibody [4]. It is a novel fragment crystallizable molecule and its binding with Fc gamma receptor IIIA (FcyRIIIA) leads to differential immune effector functions promoting a response in otherwise so called ‘cold tumors’ [5]. The multi-functionality of the CTLA-4 inhibitor is considered responsible for the differential efficacy and toxicity profile seen with the BOT [4, 5].

As novel and more efficacious drugs are developed, there is a need to revisit the tumor regression grading (TRG) systems which assess histopathological response. The Mandard-TRG system developed in 1994 in France based on features of regression pattern seen in response to chemoradiotherapy in patients with esophageal cancer, is most commonly used [6]. It roughly estimates the percentage of residual, viable cancer within the often fibrotic tumor bed. However, with the advent of immunotherapy for melanoma and lung cancer, various modifications and quantification schema have been proposed [7]. Yet these still focus more on the quantification of the residual cancer cells in the regression bed, and less on the location of these cells and the pattern of responses observed.

It is well known that for mismatch repair proficient/microsatellite stable (pMMR/MSS) colon and rectal cancer, immunotherapy historically has shown limited efficacy [8]. While the ‘adenoma-carcinoma sequence’ explaining the general development of most colorectal cancers is well described, little is known about regression patterns in response to checkpoint blockade [9].

**Fig. 1:**
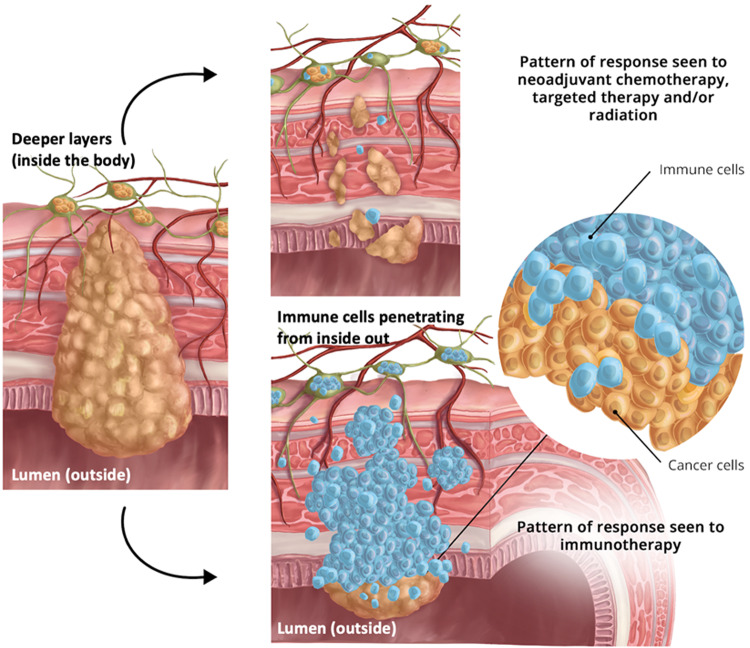
‘Inside-out’ (serosa-to-mucosa) regression pattern of response seen in patients with colon and rectal cancer receiving botensilimab plus balstilimab in the neoadjuvant setting. As shown, compared to traditional responses seen with chemotherapy, targeted therapy and/or radiation, the residual proportion of the tumor cells are all confined to the luminal surface with immunotherapy. This can be best described as a wave or tsunami of immune cells infiltration and subsequent cancer cell death.

**Fig. 2:**
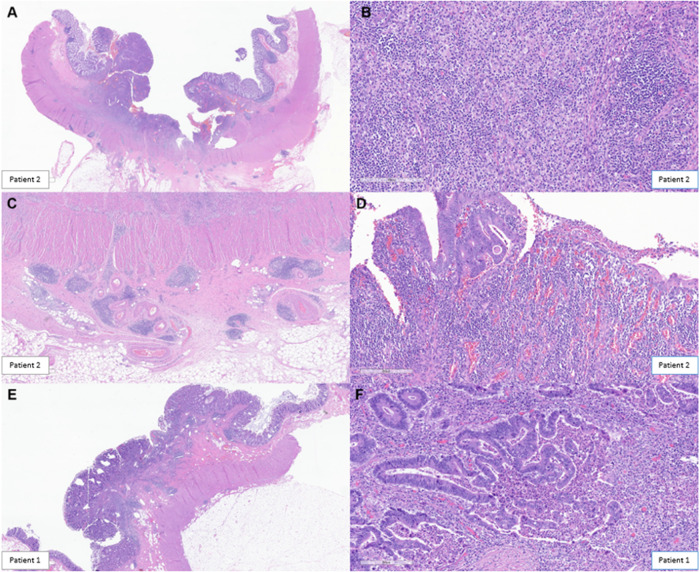
Histopathological Rreview outlining key features noted post-treatment with immunotherapy. Histopathologic review was based on surgical specimens of the tumors post-treatment that were entirely submitted for the two patients showing marked therapeutic response. Microscopically, a dense mixed inflammatory infiltrate was identified surrounding the tumor mass in Patient 2 (**A**). The infiltrate was lymphoplasmacytic-rich but also contained frequent macrophages (some foamy), occasional multinucleated giant cells, eosinophils, and neutrophils (**B**). Tertiary lymphoid structures (TLS) or Crohn like reaction was commonly seen at the periphery (**C**). Neovascularization was a prominent feature in most tumor beds, and granulation tissue predominated along the luminal surface (**D**). A similar response was noted in Patient 1. Rather than haphazardly arranged within dense fibrosis, as is often seen with neoadjuvant chemotherapy, viable tumor was often superficially oriented near the luminal surface within the tumor center, with dense inflammation surrounding the periphery and comprising most of the grossly identifiable tumor bed (**E**). Residual tumor glands often demonstrated evidence of ongoing destruction with incomplete lumens and frequent luminal microabscesses (**F**).

Herein, we report and comprehensively characterize an *inside-out* (serosa-to-mucosa) pattern of responses observed with neoadjuvant BOT alongside balstilimab (BAL; an anti-PD-1 antibody) in patients with pMMR/MSS colon and rectal cancer (Figs. 1, 2). The histologic pattern of cancer cell death by the immune cells with the BOT/BAL combination within weeks of immunotherapy has not been described previously. Furthermore, using spatial biology we reveal mechanisms of actions not previously known about BOT.

## Results

We herein report detailed analyses on the pattern of exceptional responses in the first 2 patients (1 with colon cancer and 1 with rectal cancer with pathologic response) to the BOT/BAL regimen seen with only one dose of BOT and two doses of BAL. Interestingly, we observe a unique histologic pattern of therapeutic response shown in Fig. 1, a pattern that is best described as ‘*inside-out’* (serosa-to-mucosa) tumor regression.

We have previously come across the initial descriptions by Cottrell and colleagues describing the regression pattern seen in their work on patients with lung cancer as ‘*outside-in*’ [10]. The ‘*outside-in*’ pattern of response infers the regression bed with the immune infiltrates typically surrounded the residual tumor foci and abutted normal background lung tissue. Conversely, as colon and rectal cancers develop inwards penetrating deeper layers of the colon wall and spreading to adjacent lymph nodes, the immunotherapy response is typified by an ‘*inside-out*’ (serosa-to-mucosa) regression pattern. As observed in Figs. 1, 2, rather than haphazardly arranged within dense fibrosis, as is often seen with neoadjuvant chemotherapy, targeted therapy or radiotherapy, viable tumor was often superficially oriented *near the luminal surface* within the tumor center, with dense inflammation surrounding the periphery and comprising most of the grossly identifiable tumor bed (i.e., regression bed as previously described) (Fig. 2A–E). The inflammation was lymphoplasmacytic-rich with frequent areas of foamy macrophages, neutrophils, and occasional eosinophils (Fig. 2B). A Crohn-like reaction, or tertiary lymphoid structures, was prominent in the deeper colonic wall layers surrounding the tumor cells (Fig. 2C).

Residual tumor glands demonstrated evidence of ongoing destruction with incomplete lumens and frequent luminal microabscesses (Fig. 2F). The infiltrating of immune cells and subsequent cancer cell death has been likened to a “wave” or “tsunami” whereby in these exceptional responders, the residual tumor is left at the tip/superficial layers of the colon in the mucosa/submucosa, and the deeper layers are spared. Both these cases prior to therapy were classified as advanced T-stage and node positive disease. Additionally, no residual cells were seen in any of the lymph nodes. Other high-risk features, for example, lymphovascular invasion (LVI), perineural invasion (PNI), or tumor budding were not seen. In its true sense, the cancer is being attacked and killed from the inside-out by the patient’s own immune cells, with less likelihood that any micrometastatic disease is left behind.

**Fig. 3:**
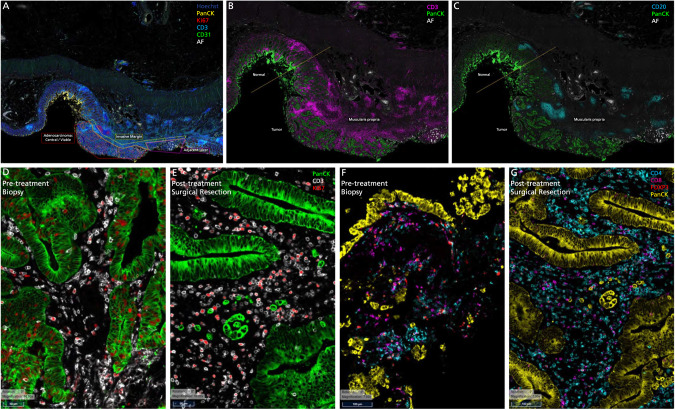
Tissue immune-microenvironment correlates assessed pre- and post-treatment with immunotherapy using RareCyte Inc. for the patient with colon cancer. **A** Residual tumor at the tip (colonic mucosa) post-treatment: Invasive margin separates normal colon from the adenocarcinoma and an ulcer. “Inside-out” (serosa-to-mucosa) pattern of regression is evident after treatment with the residual tumor only remaining within the mucosa and superficial submucosa; final path pT1aN0, as opposed to earlier at least T2N1 tumor in this first patient *(Patient 1)*. **B**, **C** Extensive immune infiltration post-treatment: The section shows extensive expansion & infiltration of CD3+ T-cells (**B**) confined to the tumor area in comparison to the adjacent normal colonic tissue after treatment. T-cells surround the tumor cells and extend deep into the muscle layer and the serosal layer. Crohn’s-like reaction is present with many CD20+ B-cells (**C**) forming follicles specifically in the tumor area & extending into the deeper muscularis propria and serosa *(Patient 1)*. **D**, **E** Immune Proliferation (comparison pre- and post-treatment): Ki67 proliferation index for immune cells is markedly increased in the post-treatment surgical resection specimen (58%) compared to the pre-treatment biopsy specimen (23%), especially for T-cells *(Patient 1)*. **F**, **G** T-cell density (comparison pre- and post-treatment): T cell density (including all T cell subsets) within the tumor increased dramatically with treatment (1398.4/mm^2^ for resection vs 190.7/mm^2^ for biopsy). Treg density also increased (411.0/mm^2^ for resection vs 56.1/mm^2^ for biopsy). However, the ratio of Treg to effector T helper cells in the tumor decreased (36% for resection vs 55% for biopsy) *(Patient 1)*.

**Fig. 4:**
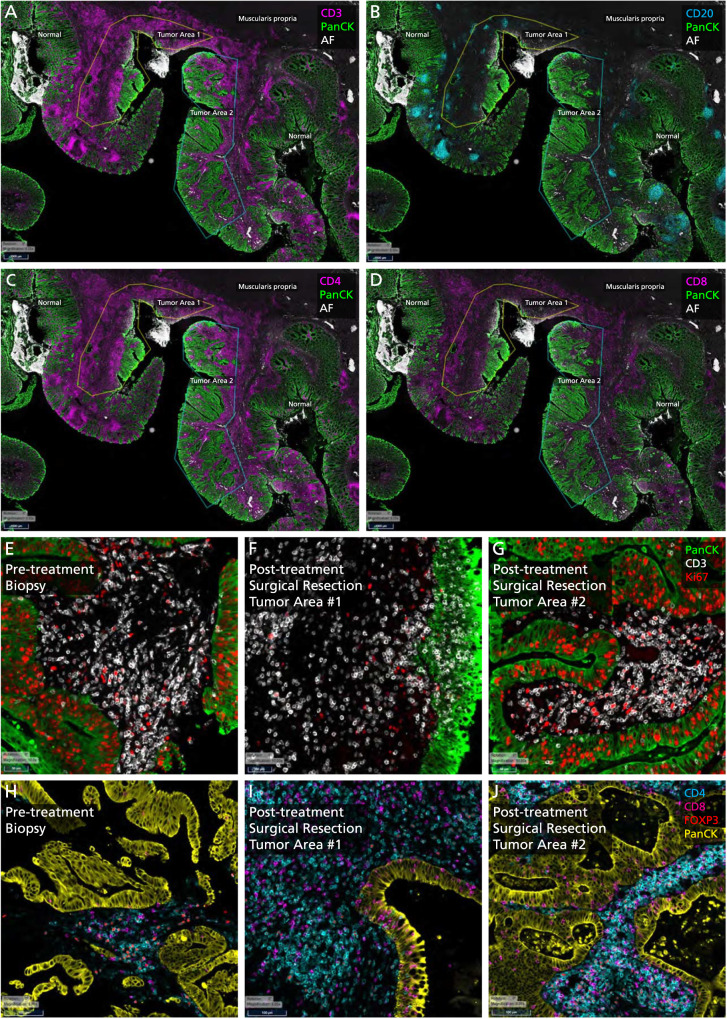
Tissue immune-microenvironment correlates assessed pre- and post-treatment with immunotherapy using RareCyte Inc. for the patient with rectal cancer. CD3+ (**A**) and CD20+ cells (**B**) in tumor microenvironment post-treatment: This section shows extensive expansion & infiltration of CD3+ T-cells (**A**) in tumor area #1 associated with tumor regression upon treatment, in comparison to tumor area #2 which shows less extensive T cell infiltration and no obvious tumor regression *(Patient 2)*. **B** shows the CD20+ B-Cells and a Crohn’s like reaction. **C**, **D** CD4+ and CD8+ cells in tumor microenvironment post-treatment: This section shows: CD4+ T-helper cells are the main immune cell type in the tumor microenvironment (area #1: 1021.8 /mm^2^; area #2: 389.3) in the post-treatment resection (**C**). Few T-helper cells are present superficially in the lamina propria of the adjacent normal colonic tissue. CD8 cytotoxic cells (CTLs) are more prevalent within the tumor area #1 (378.4 /mm^2^) compared to tumor area #2 (134.2 /mm^2^) consistent with tumor regression in the former but not latter (**D**). CTLs are markedly increased and clustered in the invasive margin (862.1 /mm^2^ and 604.1 /mm^2^) for areas 1 and 2, respectively protecting the normal deeper tissue from invasion by tumor cells *(Patient 2)*. **E**–**G** Immune Proliferation (comparison pre- and post-treatment): Ki67 proliferation index for immune cells is markedly increased in post-treatment surgical resection tumor area #2 (35%, right, mucosal adenocarcinoma—(**G**)) compared to area #1 (20%, center, deep invasive adenocarcinoma associated with tumor regression—(**F**)) and to the pre-treatment biopsy specimen (16%, **E**) *(Patient 2)*. Note: The regions of interest (ROIs) Tumor Area#1 and Tumor Area #2 are illustrated in (**A**, **B**). **H**, **I** and **J** T Cell Density: T cell density after treatment is increased within the tumor area #1 (1267.7/mm^2^, deep invasive adenocarcinoma associated with tumor regression, **I**) but not tumor area #2 (543.6/mm^2^, mucosal adenocarcinoma, **J**) compared to the pre-treatment biopsy specimen (483.9/mm^2^, **H**). Treg density increased with treatment (236.5/mm^2^ for area #1, 201.3/mm^2^ for area #2 vs 132.5/mm^2^ for biopsy). However, the ratio of Treg to effector T helper cells in the tumor decreased for area #1 and increased for area #2 (27% and 49% for resection vs 34% for biopsy) *(Patient 2)*. Note: The regions of interest (ROIs) Tumor Area #1 and Tumor Area #2 are illustrated in (**A**, **B**).

**Fig. 5:**
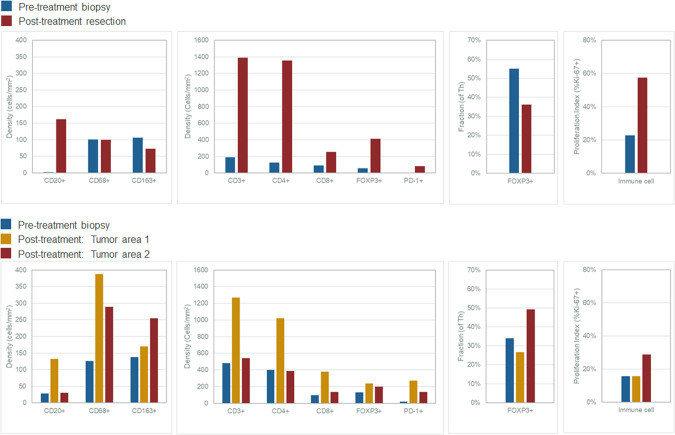
Quantitative changes in immune cell populations noted pre- and post-treatment with immunotherapy. **A** Graphs showing changes in immune cell populations in Patient 1 with colon cancer (Top Panel - Patient 1): Graphs showing changes in immune cell populations in Patient 1: All B and T cell densities increase in the resection samples (red) compared to the pre-treatment biopsy (blue) while macrophage populations decreased. Proportion of Th (FOXP3+) cells that are Tregs decreased in the resection samples compared to the pre-treatment biopsy. Finally, the proportion of immune cells that are proliferating (Ki67+) is increased in the resection samples compared to the pre-treatment biopsy. **B** Graphs showing changes in immune cell populations in Patient 2 with rectal cancer (Lower Panel - Patient 2): All B and T cell densities increase in tumor area #1 of resection samples (yellow) compared to tumor area #2 (red) and pre-treatment biopsy (blue); macrophage populations increased in both tumor areas with treatment. Proportion of Th cells that are Treg (FOXP3+) decreased in tumor area #1 and increased in tumor area #2 of the resection samples compared to the pre-treatment biopsy. Finally, the proportion of immune cells that are proliferating is increased in tumor area #2 of the resection samples compared to the pre-treatment biopsy.

Analyses of the biopsy and surgical samples pre- and post-immunotherapy shows not only a significant increase but also a diverse array of immune cells (Figs. 3–5). The pattern and trends are nearly identical in more than one instance. Figure 3A–G outline and characterize the changes seen in the first patient who had a major pathological response (*Patient 1*). Figure 4A–J outline the changes pre- and post-therapy shown in parallel in the second patient who had a major pathological response (*Patient 2*). Of note, we demonstrate intra-tumoral microenvironment heterogeneity in terms of immune response. As such, for the second case, we separated the deeper, inflammatory zone of regression (Tumor Area #1) from the more superficial area with residual tumor (Tumor Area #2) to analyze some of the changes seen in these distinct regions (Fig. 4A, B). Table 1 and Fig. 5 comprehensively outline changes in the immune repertoire in both the patients.

**Table 1:** Comparison pre- and post-treatment of the immune repertoire changes seen in the 2 patients.

Patient	Patient 1		Patient 2		
Treatment	Baseline Biopsy	Surgical Resection	Baseline Biopsy	Surgical Resection	Surgical Resection
Location	Ascending colon		Rectum		
Pathology	Tubular Adenoma	Invasive Adenocarcinoma	Invasive Adenocarcinoma	Adenocarcinoma	Adenocarcinoma
Area/ROI name	Total tumor	Total tumor	Total tumor	Tumor area 1 (deep invasive + ulcer)	Tumor area 2 (mucosal)
Area (mm^2^)	12.06	6.01	5.321	10.57	14.9
Analyte	Value	Value	Value	Value	Value
CD20+ cell density	2.2	161.4	28.2	132.5	30.2
CD68+ cell density	100.3	99.8	125.9	387.9	288.6
CD163+ cell density	106.6	73.2	138.1	170.3	255.0
CD3+ cell density	190.7	1389.4	483.9	1267.7	543.6
CD4+ cell density	126.0	1356.1	402.2	1021.8	389.3
CD8+ cell density	89.0	252.9	94.9	378.4	134.2
FOXP3+ cell density	56.1	411.0	132.5	236.5	201.3
FOXP3+ fraction	55%	36%	34%	27%	49%
PD-1+ cell density	6.2	81.5	17.9	274.4	134.2
PD-L1 CPS score	58%	70%	<1%	10%	30%
PD-L1 TPS score	<1%	0%	0%	0%	<1%
Immune Proliferation Index	23%	58%	16%	20%	35%

Using PathViewer software, pathologist draws regions of interest (ROI(s)) corresponding to tumor-bearing regions of the image, then counts cells based on visual inspection. The software reports region of interest (ROI) area allowing cell density calculation. As noted, the immune cells are noted to have increased several folds. Furthermore, intra-tumoral heterogeneity and changes can also be appreciated as noted for the 2 Tumor Areas #1 and #2 for Patient 2.

From a mutational and next generation sequencing standpoint, no molecular alterations appeared to explain the exceptional response that was noted. The first pMMR/MSS tumor was in the ascending colon and was *RAS-RAF-*wildtype with alterations in *TP53*, *CTNNB1*, and a tumor mutational burden (TMB) of 6.4 Mut/Mb, whereas the second tumor was a rectal cancer above the peritoneal reflection (candidate for upfront surgery), *KRAS-G12V* mutant, and with truncating alterations in *APC* gene and a TMB of 4.7. Final pathology in both cases was ypT1N0MX. Both cases as noted earlier were advanced and node positive prior to starting treatment (specifically patient 2 with rectal cancer on an MRI had T3bN2 tumor; while patient 1, since it is a CT scan, we can with limitations say advanced stage/node positive (T2N1)).

## Discussion

Herein, we provide the first comprehensive histologic characterization and the activity of this specific BOT/BAL regimen in patients with pMMR/MSS colon and rectal cancer in the neoadjuvant setting. Previously, work by Chalabi et al. reported in the initial landmark NICHE-1 clinical trial included patients with both pMMR/MSS and dMMR/MSI-High colon cancers using ipilimumab and nivolumab (IPI/NIVO) [11]. Subsequent work in the NICHE-2 trial focused on the latter subset of dMMR/MSI-High colon cancers. This phenomenal curative activity of immunotherapy was also replicated in dMMR/MSI-High rectal cancers by Cercek et al. [12]. However, there has been limited progress in the utilization of immunotherapy for patients with pMMR/MSS colon and rectal cancers.

Thus far, the BOT/BAL regimen has shown promising activity in patients with metastatic refractory pMMR/MSS colon and rectal cancers, and especially those without untreated liver metastases [4]. Our work is unique and clinically of value since we are showing (a) activity of an immunotherapeutic agent in pMMR/MSS colon and rectal tumors, and more importantly (b) the pattern of regression and cancer cell death observed has important potential clinical implications. The observation about the pattern of response is that all the tumor is left at near the luminal surface and regressed elsewhere in the whole surgical specimen. With confirmation in a larger cohort and/or a randomized clinical trial, these patients potentially could be spared the toxicity of adjuvant chemotherapy. Novel minimal residual disease tools like circulating tumor DNA (ctDNA) could be used to direct adjuvant therapy as is being done in some of the ongoing trials. Additionally, this type of immune response could be pathognomonic for how the immune system may respond to a tumor once activated and may also explain the phenomenon of pseudo-progression. These findings may have implications for how future clinical trials are designed in the neoadjuvant setting.

The purpose of adjuvant therapy is to eradicate any micrometastatic disease remaining. Currently, for advanced colon and rectal cancer patients who undergo surgery and found to be node positive (stage III) or high risk stage II, 3–6 months of adjuvant chemotherapy is recommended [13]. Neoadjuvant immunotherapy with this novel regimen could obviate the need for chemotherapy and/or increase the proportion of patients who can be cured if it can eradicate micro-metastatic disease “*inside-out*” since residual disease may be limited to superficial bowel wall layers on the final surgical specimen. This observation for the time being remains hypothesis generating and requires confirmation in larger studies.

These preliminary results pave the way for further development of this highly active BOT/BAL regimen for patients with pMMR/MSS colon and rectal cancers, with plans to bring it earlier in the journey of a patient to the neoadjuvant setting. Limitations of this report include the descriptive nature of the findings and no direct comparison with other drugs in a similar cohort. Furthermore, it is possible that the pattern of response may not be unique to BOT/BAL, but generalizable to various immunotherapies given in the neoadjuvant setting. This, however, has not been officially reported with other regimens and trials in this setting. The comprehensive analyses using the spatial testing using the RareCyte Inc. platform adds further to our understanding of the immune microenvironment and how this regimen alters cell type within the tumor-stromal interface.

Finally, this work also highlights the limitations of the traditional response assessment systems, not just the mere quantification, as the specific pattern of therapeutic response generated may dictate how best to histologically assess the post-therapeutic specimen. While the “outside-in” pattern is relevant to primary parenchymal tumors as described by Cottrell et al., as well as potentially for metastatic sites, the inside-out (serosa-to-mucosa) pattern characterizes the immune response for primary sites of the luminal cut such as colon or rectum that arise from a polyp and penetrate the deeper layers of the walls of the lumen [10]. It would be important to not only note the percentage of residual cancer cells, but also where are they located, and how has the treatment downstaged the cancer (e.g., a clinically staged T3bN2 tumor before treatment, which is now a pT1N0 on final surgical pathology after neoadjuvant immunotherapy would be very different from an alternate scenario where residual cells are far few but scattered and/or with positive margins (Fig. 1)).”

Given the novelty of these findings and first reports of activity of BOT in the neoadjuvant setting we decided to disseminate this albeit in the first 2 exceptional responders since there is interest in this drug/combination in patients with pMMR/MSS colorectal cancers. Many trials are in development in this space for both patients with colon and rectal cancers, and the analyses/observations would be of value for other researchers. The NEST study is rapidly accruing and nearing completion (ClinicalTrials.gov Identifier: NCT05571293). More analyses on the larger cohort once completed would add further to our understanding of neoadjuvant immunotherapy responses in general, and to the mechanisms of actions of BOT.

## Methods

The ‘inside-out’ (serosa-to-mucosa) pattern of response was observed and analyzed in patients with brisk major pathologic responses to the BOT/BAL regimen as part of the investigator-initiated trial (IIT; ClinicalTrials.gov Identifier: NCT05571293: “Novel Exploratory Study to Test combination of Botensilimab and Balstilimab Immunotherapy in Patients with Resectable Colorectal Cancer (NEST-1)”) [14]. NEST-1 was initiated based on the clinical trial by Chalabi *et al*. called NICHE-1 (Neoadjuvant Immune Checkpoint Inhibition and Novel IO Combinations in Early-stage Colon Cancer) [11]. As part of the NEST-1 trial, patients received one fixed dose of 75 mg of BOT and two doses of 240 mg of BAL 2 weeks apart; with the first dose of BAL the same day as the BOT (BOT/BAL regimen). Like the NICHE-1 study, patients with non-metastatic locally advanced colorectal cancers could be enrolled. We allowed patients with rectal cancers specifically if their treatment plan was upfront surgery and no need for neoadjuvant chemotherapy or radiation. Since we expect patients with dMMR/MSI-High tumors to respond well to checkpoint blockade, the focus of this study was predominantly on pMMR/MSS colorectal cancers. Thus, while patients with dMMR/MSI-High colorectal cancers were allowed to enroll, we limited enrollment to no more than 25% of the cohort; 75% of our planned pilot study had to be patients with pMMR/MSS colon and rectal cancers. Detailed inclusion and exclusion criteria are listed on clinicaltrials.gov: NCT05571293 [14]. Patients could proceed to curative-intent surgical resection at least 1 week after the second dose of BAL.

Tissue immune-microenvironment correlates were assessed at baseline as well as in the surgical specimen. For the latter, RareCyte Inc. (Seattle WA), performed a 13-marker immune-oncology panel that was developed by the company to test the pre- and post-treatment colon and rectal cancer samples on a single paraffin-embedded slide simultaneously at 20X using the Orion instrument (Figs. 3, 4, 5, Supplementary Table 1) [15, 16]. The RareCyte platform was chosen based on work published in this space by other researchers using similar platform with the company, prior collaborations, the ability to use formalin fixed specimens, efficiently evaluate the immune environment requiring minimal tissue input and providing rapid real-time turnaround [17]. Briefly, FFPE sections (5 μm) on glass slides were baked, dewaxed with xylene, antigen-retrieved (pH 8.5), quenched to reduce endogenous tissue fluorescence, then stained manually with the RareCyte 13-plex Immuno-oncology panel of fluorescent probes for the following biomarkers: Nucleus (Hoechst), CD3, CD4, CD8, CD20, CD31, CD68, CD163, FOXP3, Ki67, PanCK, PD-1, PD-L1 (Supplementary Table 1). The stained slides were imaged on the Orion instrument, processed to TIFF images, and loaded into pathology image review software (PathViewer software). Tumor ROI(s) were identified by Pathologist review of H&E and IF images to determine tumor area(s). The samples were then analyzed via pathologic assessment and a quantitative image analysis pipeline for deep interrogation of the immune spatial environment. This included tumor immune cell subset percentages and tumor proliferation index, each of which was reviewed by a pathologist for amongst other things, tumor hot/cold assessment. Results were compiled and presented with descriptive statistics summarizing the intra- and inter-patient results and trends/patterns observed via both analysis methods. Sequencing on the tumor samples was performed using our inhouse TruSight Oncology 500 (TSO 500, Illumina Inc. platform) that includes DNA, RNA, microsatellite instability assessment through next generation sequencing, as well as information about tumor mutational burden (TMB).

### Supplementary information

Supplementary Table 1

## Data Availability

All data generated or analyzed during this study are included in this published article and the supplementary files.

## References

[1] Le Saux O, Lounici Y, Wajda P, Barrin S, Caux C, Dubois B (2021). Neoadjuvant immune checkpoint inhibitors in cancer, current state of the art. Crit Rev Oncol Hematol.

[2] Marron TU, Galsky MD, Taouli B, Fiel MI, Ward S, Kim E (2022). Neoadjuvant clinical trials provide a window of opportunity for cancer drug discovery. Nat Med.

[3] Chalabi M. The promise of neoadjuvant immunotherapy across solid tumors. 2023. https://meetings.asco.org/2023-asco-annual-meeting/15057?presentation=225720#225720 (Accessed 18 Aug 2023).

[4] El-Khoueiry AB, Fakih M, Gordon MS, Tsimberidou AM, Bullock AJ, Wilky BA (2023). Results from a phase 1a/1b study of botensilimab (BOT), a novel innate/adaptive immune activator, plus balstilimab (BAL; anti-PD-1 antibody) in metastatic heavily pretreated microsatellite stable colorectal cancer (MSS CRC). J Clin Oncol.

[5] Delepine C, Levey D, Krishnan S, Kim K-S, Sonabend A, Wilkens M (2022). 470 Botensilimab, an Fc-enhanced CTLA-4 antibody, enhances innate and adaptive immune activation to promote superior anti-tumor immunity in cold and I-O refractory tumors. J Immunother Cancer.

[6] Mandard AM, Dalibard F, Mandard JC, Marnay J, Henry-Amar M, Petiot JF (1994). Pathologic assessment of tumor regression after preoperative chemoradiotherapy of esophageal carcinoma clinicopathologic correlations. Cancer.

[7] Tetzlaff MT, Messina JL, Stein JE, Xu X, Amaria RN, Blank CU (2018). Pathological assessment of resection specimens after neoadjuvant therapy for metastatic melanoma. Ann Oncol J Eur Soc Med Oncol.

[8] Le DT, Uram JN, Wang H, Bartlett BR, Kemberling H, Eyring AD (2015). PD-1 blockade in tumors with mismatch-repair deficiency. N Engl J Med.

[9] O’Brien MJ, Gibbons D (1996). The adenoma-carcinoma sequence in colorectal neoplasia. Surg Oncol Clin N Am.

[10] Cottrell TR, Thompson ED, Forde PM, Stein JE, Duffield AS, Anagnostou V (2018). Pathologic features of response to neoadjuvant anti-PD-1 in resected non-small-cell lung carcinoma: a proposal for quantitative immune-related pathologic response criteria (irPRC). Ann Oncol J Eur Soc Med Oncol.

[11] Chalabi M, Fanchi LF, Dijkstra KK, Van den Berg JG, Aalbers AG, Sikorska K (2020). Neoadjuvant immunotherapy leads to pathological responses in MMR-proficient and MMR-deficient early-stage colon cancers. Nat Med.

[12] Cercek A, Lumish M, Sinopoli J, Weiss J, Shia J, Lamendola-Essel M (2022). PD-1 blockade in mismatch repair-deficient, locally advanced rectal cancer. N Engl J Med.

[13] Grothey A, Sobrero AF, Shields AF, Yoshino T, Paul J, Taieb J (2018). Duration of adjuvant chemotherapy for stage III colon cancer. N Engl J Med.

[14] ClinicalTrials.gov. Novel Exploratory Study to Test Combination of Botensilimab and Balstilimab Immunotherapy in Resectable Colorectal Cancer Patients. clinicaltrials.gov, 2023 https://clinicaltrials.gov/study/NCT05571293 (Accessed 16 Aug 2023).

[15] Lin J-R, Chen Y-A, Campton D, Cooper J, Coy S, Yapp C (2023). High-plex immunofluorescence imaging and traditional histology of the same tissue section for discovering image-based biomarkers. Nat Cancer.

[16] Anderson AN, Gibbs SL (2023). Shooting for multiplexed pathology with Orion. Nat Cancer.

[17] Lin J-R, Wang S, Coy S, Chen Y-A, Yapp C, Tyler M (2023). Multiplexed 3D atlas of state transitions and immune interaction in colorectal cancer. Cell.

