# Supplementary material for: Neoadjuvant botensilimab plus balstilimab response pattern in locally advanced mismatch repair proficient colorectal cancer
Source: Oncogene. 2023 Sep 21;42(44):3252–9. doi: 10.1038/s41388-023-02835-y (PMC10611560; doi:10.1038/s41388-023-02835-y)
Supplement: Supplementary file 1 — Supplementary Table 1 [file 41388_2023_2835_MOESM1_ESM.docx]

**Supplementary Table 1:** 13-marker immune-oncology panel developed by RareCyte Inc. Seattle WA to test the pre- and post-treatment colon and rectal cancer samples in one staining round and one imaging round at 20X using the Orion instrument.

| ***Analyte*** | ***Definition*** |
| --- | --- |
| ***CD20+ cell density*** | CD20+ count (primarily B cells) per mm^2^ of tumor area |
| ***CD68+ cell density*** | CD68+ count (primarily Macrophages) per mm^2^ of tumor area |
| ***CD163+ cell density*** | CD163+ count (primarily Macrophages) per mm^2^ of tumor area |
| ***CD3+ cell density*** | CD3+ count (primarily T cells) per mm^2^ of tumor area |
| ***CD4+ cell density*** | CD4+ count (T helper cells [Th] and some macrophages) per mm^2^ of tumor area |
| ***CD8+ cell density*** | CD8+ count (primarily cytotoxic T lymphocytes [CTL]) per mm^2^ of tumor area |
| ***FOXP3+ cell density*** | FOXP3+ count (primarily regulatory T cells [Treg]) per mm^2^ of tumor area |
| ***FOXP3+ fraction*** | FOXP3+ as a percentage of Th cells (CD3+ cells minus CD8+ cells) |
| ***PD-1+ cell density*** | PD-1+ count (primarily a subset of T cells) per mm^2^ of tumor area |
| ***PD-L1 CPS score*** | Combined Positive Score: PD-L1+ immune and tumor cells as a percentage of total cells (tumor, immune, stromal) |
| ***PD-L1 TPS score*** | Total Proportional Score: PD-L1+ tumor cells as a percentage of total tumor cells |
| ***Immune Proliferation Index*** | Ki67+ immune cells as a percentage of total immune cells |
| ***Tumor Hot/Cold*** | Extent and inflammatory status of T-cell tumor infiltration; high (Hot) or low (Cold) |
